# AI-based analysis of climatic and air pollution determinants of dog bite incidence

**DOI:** 10.3389/fvets.2025.1731641

**Published:** 2026-02-13

**Authors:** Sneha Gautam, Aron Rodrick Lakra, N. S. Athish, Lazarus Godson Asirvatham, Bairi Levi Rakshith, Chang-Hoi Ho, Vibhanshu Vaibhav Singh, Vincent Sam Jebadurai, Lavudiya Ramesh Babu

**Affiliations:** 1 Division of Civil Engineering, Karunya Institute of Technology and Sciences, Coimbatore, Tamil Nadu, India; 2 Department of Climate and Energy Systems Engineering, Ewha Womans University, Seoul, Republic of Korea; 3 Division of Computer Science and Engineering, Karunya Institute of Technology and Sciences, Coimbatore, Tamil Nadu, India; 4 Division of Artificial Intelligence and Machine Learning, Karunya Institute of Technology and Sciences, Coimbatore, Tamil Nadu, India; 5 Division of Mechanical Engineering, Karunya Institute of Technology and Sciences, Coimbatore, Tamil Nadu, India; 6 Centre for Research in Material Science and Thermal Management (CRMS &TM), Karunya Institute of Technology and Sciences, Coimbatore, Tamil Nadu, India; 7 Division of Biotechnology, Karunya Institute of Technology and Sciences, Coimbatore, Tamil Nadu, India

**Keywords:** artificial intelligence, climate change, dog bite incidents, environmental dynamics, pollution parameters, public health implications

## Abstract

Dog bite incidents are an emerging public health concern that may be influenced by changing environmental conditions. This study investigated the relationship between meteorological variables (maximum temperature and relative humidity) and dog bite incidence across five Indian states: Bihar, Karnataka, Punjab, Telangana, and Uttar Pradesh. The role of key air pollutants, including formaldehyde, nitrogen dioxide, sulfur dioxide, and ozone, was also examined. Statistical analyses showed that maximum temperature (*p* = 0.0014) and relative humidity (*p* = 0.0252) were significantly associated with dog bite incidence, with higher temperatures associated with increased incidence and higher humidity with reduced incidence. Principal component analysis (PCA) revealed no apparent clustering or dominant trend in environmental factors, indicating that temperature and humidity alone do not sufficiently explain dog bite variability across regions. Correlation analysis across monthly data demonstrated a strong overall positive association with maximum temperature (*r* = 0.84), although short-term annual trends show nonlinear fluctuations influenced by additional contextual factors. To predict dog bite risk, an artificial intelligence model (H2O XGBoost) was developed, achieving 87% accuracy and a mean absolute percentage error of 9.6%. This study highlights the importance of localized environmental interpretation and region-specific variability, contributing to understanding the ecological determinants of animal-related injuries and supports Sustainable Development Goals 3 (good health and well-being), 11 (sustainable cities and communities), and 13 (climate action) by informing strategies for safer and more resilient urban environments.

## 1 Introduction

Dog bites constitute a significant global public health concern, affecting both high-income and low- and middle-income countries, and leading to substantial medical, social, and economic burdens (1). In low- and middle-income countries, dog bites account for about 85% of all animal bite-related injuries and deaths, making them the predominant source of such incidents (2). On average, 25.7 dog bite cases are reported per 1,000 individuals annually, though the actual number is likely higher due to widespread underreporting (3). In high-income countries such as the United States, an estimated 4.5 to 4.7 million dog bite incidents occur each year, with around 800,000 requiring medical treatment (4, 5). In the United Kingdom, about 740 cases per 100,000 individuals are reported annually (6). Although advances in timely medical care and systematic vaccination programs for animals have reduced fatality rates, dog bites continue to pose serious risks, particularly to children and elders. Globally, ~40% of dog bite victims are under the age of 15, representing a physically and behaviorally vulnerable population (7, 8). One of the most serious consequences of dog bites is rabies a zoonotic disease that remains a persistent global health threat (9). Rabies is responsible for about 59,000 deaths annually, with over 99% of cases resulting from dog bites (7). More than 150 countries are affected by rabies, with the highest burdens occurring in Asia and Africa (10, 11). India alone accounts for nearly 36% of global rabies-related deaths, emphasizing the magnitude of the problem in the region (6, 12–14).

While statistical data emphasize the magnitude and severity of dog bite incidents, understanding the underlying causes is equally critical for effective prevention. Novack et al. (15) identified inconsistent human-animal interactions, inadequate training, and densely populated living environments as key contributors to dog aggression. Yeh et al. (16) demonstrated the role of sociodemographic factors in influencing bite risk. Behavioral factors, such as the victim’s age, provoking behavior, and contextual triggers, have also been noted by Reisner et al. (17), Caffrey et al. (18), and Tenzin et al. (19). Similarly, Taylor et al. (20) reported that ordinary interactions such as playing with or approaching dogs can lead to unintentional provocation. Asokan et al. (21) observed a seasonal trend, noting that dog bites were more prevalent during warmer months than during colder months. Hall et al. (22) further highlighted the influence of environmental factors, including stray dog populations and socio-cultural dynamics, on the frequency of dog bite incidents. Other studies have shown that ecological stressors, owner behavior, early-life socialization, and the pressures of urban living can collectively modulate aggressive tendencies in dogs and contribute to increased bite incidence (23, 24).

Most previous studies have focused on immediate behavioral, demographic, or seasonal factors, with limited attention paid to broader environmental influences. It should be noted that Milligan (25) and Waiblinger et al. (26) highlighted the importance of temperature and rainfall variability, as well as habitat fragmentation, in influencing animal aggression. Sweden et al. (27) also indicated that ecosystem degradation, increased animal movement, and territorial responses could contribute to greater aggression. Chen et al. (28) found that specific environmental conditions, such as low temperatures, high humidity, and poor air quality, can raise stress levels and subsequently increase the likelihood of aggressive behavior.

Based on these insights, the present study seeks to address a critical knowledge gap by examining how environmental stressors, particularly weather and air pollution, affect the incidence of dog bites in India. The primary objective is to analyze the relationship between dog bite incidence and key environmental variables, including temperature, relative humidity, formaldehyde (HCHO), nitrogen dioxide (NO_2_), sulfur dioxide (SO_2_), and ozone (O_3_). This study employs geographic information systems (GIS), principal component analysis (PCA), and XGBoost modeling algorithms across five Indian states (Bihar, Punjab, Uttar Pradesh, Karnataka, and Telangana). It is worth noting that the goals of this study align with multiple United Nations Sustainable Development Goals (SDGs). By focusing on a neglected zoonotic disease, the study contributes to SDG 3 (good health and well-being). Its emphasis on urban ecology, stray animal population management, and injury prevention supports SDG 11 (sustainable cities and communities). Furthermore, this study provides novel insights into the interactions between climate variability, air pollution, and injury risk, thereby contributing to SDG 13 (climate action). This study advances scientific understanding and facilitates the development of localized, evidence-based public health strategies that are responsive to India’s ecological diversity and socio-cultural complexity.

## 2 Materials and methods

**Figure 1:**
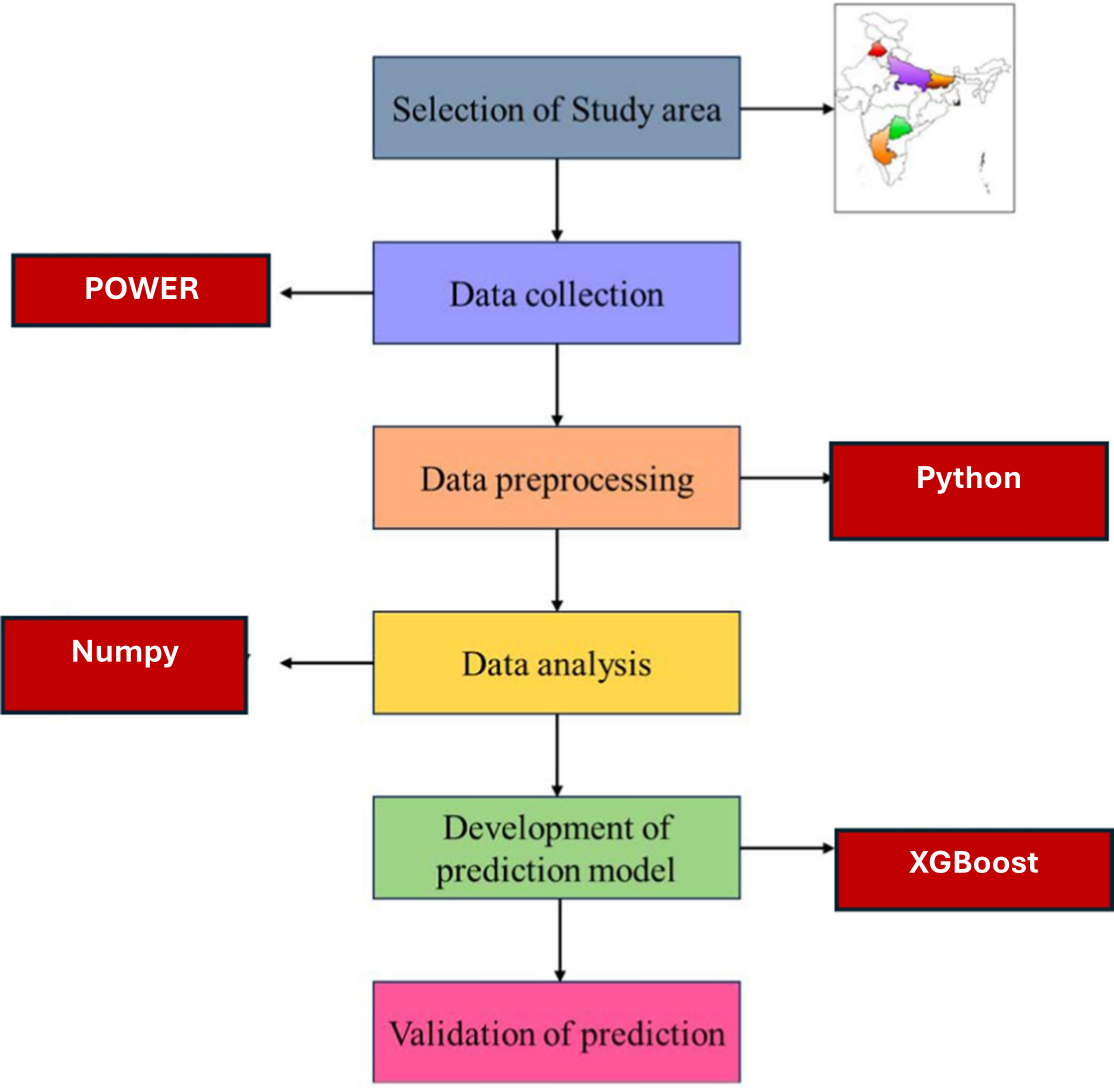
Workflow of the study.

This paper adopts a structured multi-stages approach to investigate the factors that have an effect on the distribution of dog bite incidents across five Indian states, and these factors are environmental variables like the temperature, humidity, and air pollutants. Figure 1 shows the complete methodological framework, which includes the selection of the study area, data collection, preprocessing and data cleaning, data analysis, development of the prediction model, and its validation. The subsequent sections provide detailed descriptions of each step involved.

### 2.1 Selection of area

The study areas were selected based on pre-defined criteria that represent a wide range of geographical, meteorological, socio-economic, and environmental conditions relevant to the research objectives. Key selection parameters were regional accessibility, variability in terrain and availability of ground truth and secondary data. These were essential for robust environmental analysis and the validation of machine learning-based predictive models. This study focuses on five Indian states that includes Bihar, Punjab, Uttar Pradesh, Karnataka, and Telangana each representing unique climate profiles, urbanization patterns and ecological dynamics.

**Figure 2:**
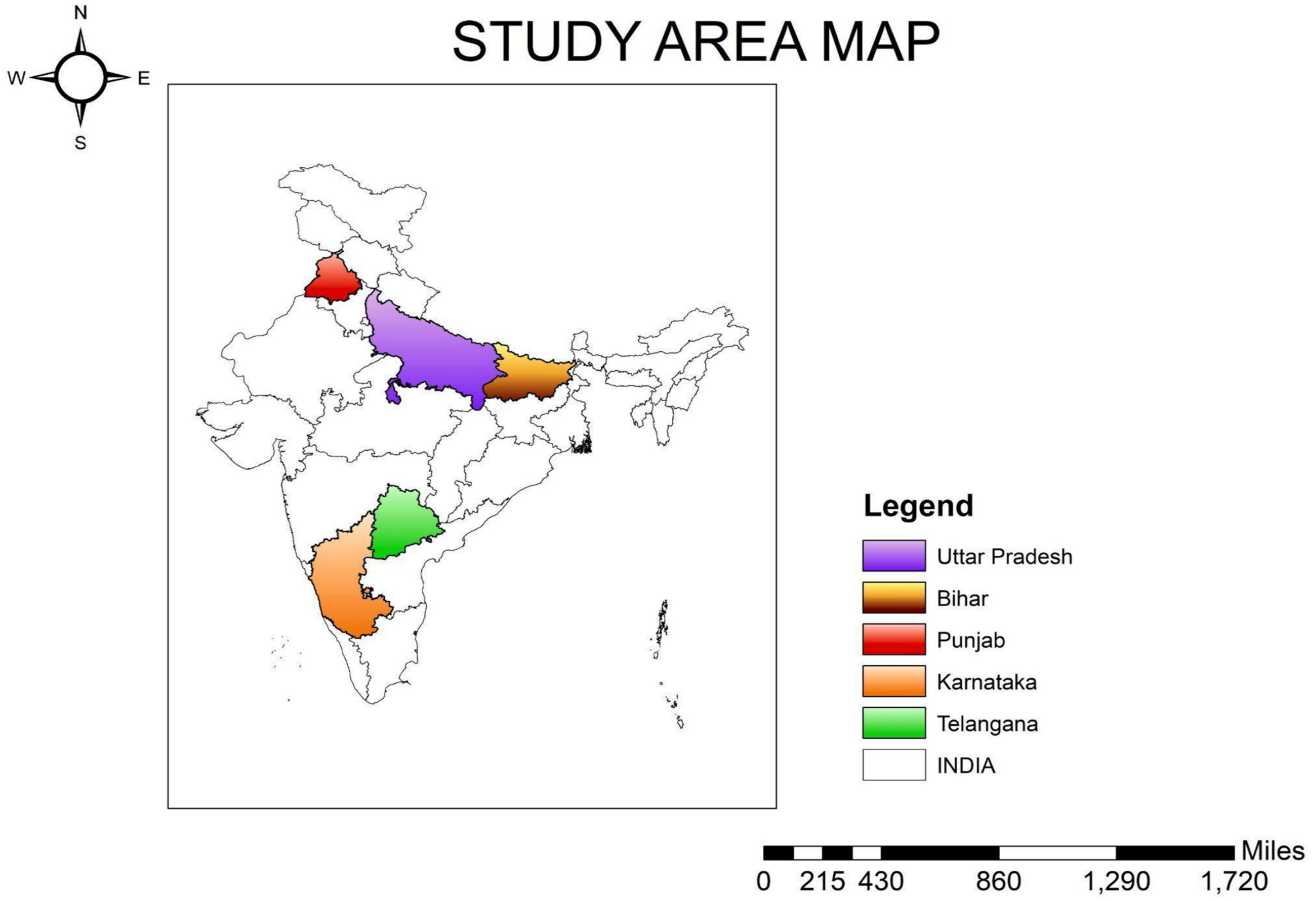
Geographical illustration of selected study areas in India.

These states were considered because they have high population density, diverse environmental exposures, and contrasting geography. Next, these states were divided into the two geographical clusters. Bihar, Punjab, and Uttar Pradesh were considered the northern region, and Karnataka and Telangana were regarded as the southern region. This facilitates understanding regional trends and variations in dog bite incidence in relation to environmental stressors such as air pollution and temperature extremes across different climate zones, as shown in Figure 2.

The study areas were chosen based on a set of multi-dimensional criteria to capture the complex relationships between environmental, social and policy factors that influence human-dog interactions. First, meteorology was prioritized as climate plays a significant role in shaping ecological dynamics and behavioral responses in both humans and animals. Second, air quality indicators such as formaldehyde (HCHO), nitrogen dioxide (NO₂), sulfur dioxide (SO₂) and ozone (O₃) were considered as they are known to be linked to physiological and neurological stress in both species. Third, socio-economic conditions and cultural norms were evaluated to understand regional differences in dog ownership, dog care and human-dog engagement. Fourth, animal welfare and stray dog control regulations were reviewed to put the public policy context for dog populations and community safety. Finally, the availability of data on dog access to basic resources such as food and water was assessed as these are basic indicators of animal welfare. These states were chosen to act as a model representing dissimilar climatic zones, population densities and urbanization levels in India as opposed to convenience sampling. Although other contextual variables like the socio-economic conditions and the municipal dog-control practices were assessed qualitatively to aid the interpretation, they were not available in all states in consistent quantitative datasets, and thus, were not used in the modeling analysis.

### 2.2 Data collection

The data collection process was multi-source and included datasets from online repositories, government portals and validated secondary sources. The variables collected for the study were demographic, environmental, and outcome-related. Meteorological data (temperature, relative humidity, precipitation) were obtained from NASA POWER-DAV (29). This source provides globally gridded meteorological data at high spatial and temporal resolution and is suitable for region-specific environmental analysis. Air pollution data for five key atmospheric constituents were obtained from NASA Giovanni (30).

The study period spans 2018 to 2023 to provide sufficient time to observe trends and associations. Selected study areas were chosen to span diverse geographical and ecological conditions, thereby increasing the robustness and generalizability of the analysis. Data on dog bite cases was obtained from the Ministry of Health and Family Welfare, Government of India, from a report published on 12th December 2023. This dataset contains state- and Union Territory-wise records of dog bite cases from 2018 to 2022 and serves as the outcome variable for the study. This resulted in a larger sample size (n = 300 observations across 5 states) for model development compared to state-wise annual values. The case records on dog bites received through the national health reports were summed monthly at the state level. The data about the meteorological (temperature and humidity) and air pollutant (HCHO, NO_2_, SO_3_, and O_3_) were obtained (as daily gridded measurements) and transformed to monthly averages to allow the temporal alignment of the dog-bite data. The analysis of all data was performed at the state level in order to ensure consistency of the spatial resolution. The temperature was recorded in ^o^C, relative humidity in percentages, and the concentration of pollutants in μg/m^3^. Satellite-based retrievals of environmental data can include negative values and yield ecological datasets. These were negative values that were not transformed, per NASA Giovanni advised protocol of handling. When combined with meteorological and air quality data, the dog bite data provide a framework to understand the impact of environmental stressors on dog-human conflict.

It should be noted that meteorological and air pollution data were collected up to 2023 to provide contextual visualization of environmental variation; however, as official dog bite case data were available only through 2022, analytical and predictive modeling were conducted using data from 2018 to 2022. The 2023 environmental data were included solely for spatial–temporal illustration.

### 2.3 Data pre-processing and handling

Before analyzing the data, the datasets were cleaned using data cleansing, normalization, and feature engineering to ensure the datasets were free of missing values, outliers, and data inconsistencies. It is essential to conduct a practical analysis of the data. Standardization procedures were employed to ensure uniformity across different datasets and study areas, including scale and format. The data were aggregated into monthly averages using pandas in Python and then into annual summaries to examine trends during the study period. Monthly-averaged values were used for correlation and significance testing to capture finer temporal variation, whereas annual summaries were generated solely for visualization of long-term patterns and trend interpretation. Dog bite records were preformatted for analysis and required minimal processing. Uniformity tests and structural validation was done to check the conformity with the environmental datasets. All pre-processing operations, including data integration, cleaning, normalization, and standardization, were performed using Python’s NumPy and pandas modules to create a single, analysis-ready dataset across spatial and temporal dimensions. All environmental datasets were standardized using z-score normalization before correlation and PCA to ensure comparability across variables with different scales. Missing values were imputed using mean substitution where applicable.

The datasets underwent a comprehensive analysis employing geographical, temporal, and multilevel techniques to derive insights and patterns. A synthesis of Geographic Information Systems (GIS), machine learning techniques, and statistical tools was employed to guarantee robustness and visualization. A spatial study of meteorological variables, specifically temperature and RH, was conducted using ArcGIS. This facilitated the visualization and examination of spatial patterns within the research sites and offered spatial context for environmental variables pertinent to human-dog interactions. Temporal study encompassed time series forecasting utilizing H2O XGBoost, a hybrid machine learning model selected for its efficacy with tabular datasets. H2O XGBoost is effective for medium-sized datasets and was employed to analyze the temporal dynamics of environmental factors and their correlation with dog bite incidence. Machine learning models were developed using pre-processed meteorological and pollutant data to identify trends and feature correlations.

A multilevel analysis was conducted to examine the hierarchical relationships among meteorological variables, air pollution indicators, and human-dog interactions. This enabled us to observe the nested linkages and their impact on the dependent variable, dog bite incidence, across both spatial and temporal dimensions. Geospatial visualization was conducted via GIS to illustrate intricate spatial relationships and regional trends, facilitating comprehension for stakeholders and policymakers. Statistical inference was performed using Python libraries: NumPy and pandas for data processing, matplotlib and mpl_toolkits.mplot3d for data visualization, and scikit-learn for machine learning. Hypothesis testing and sensitivity analysis were used to assess the significance of the observed connections. Pearson correlation coefficients and associated *p*-values were computed to examine the association between annual dog bite counts and monthly average temperature data from 2018 to 2023. The correlations identified in the data were statistically confirmed using the pearsonr function from the scipy.stats package. Pearson correlation coefficients were computed using paired monthly dog-bite case counts and monthly averaged environmental variables across the full study period. The SciPy pearsonr function was used to calculate correlation coefficients and corresponding two-tailed *p*-values to assess statistical significance.

### 2.4 Prediction modeling

The prediction phase involves developing, training, and evaluating machine learning models to predict dog bite incidents based on historical environmental and meteorological data. This phase uses established libraries such as Scikit-learn, XGBoost, and H2O.ai. To prepare the data for modeling, missing values are handled using scikit-learn’s SimpleImputer class.impute module. This ensures data integrity by imputing missing values using mean, median or mode depending on the variable distribution. The data is then split using the train_test_split function from scikit-learn, 80% for training and 20% for testing on temporal order to preserve chronological progression. Model performance was evaluated using H2O’s regression accuracy metric and mean absolute percentage error (MAPE), comparing predicted values with observed dog-bite counts in the test set. The prediction model uses XGBoost, an advanced gradient-boosting framework optimized for regression. Specifically, the XGBRegressor class is used to train the model on the selected input variables. The study uses the H2O.ai platform to build scalable and efficient models that can learn complex non-linear relationships in the data. This hybrid approach combining H2O and XGBoost delivers high predictive power, computational efficiency, and scalability.

## 3 Results

### 3.1 Geospatial distribution of temperature (T) across study areas

**Figure 3:**
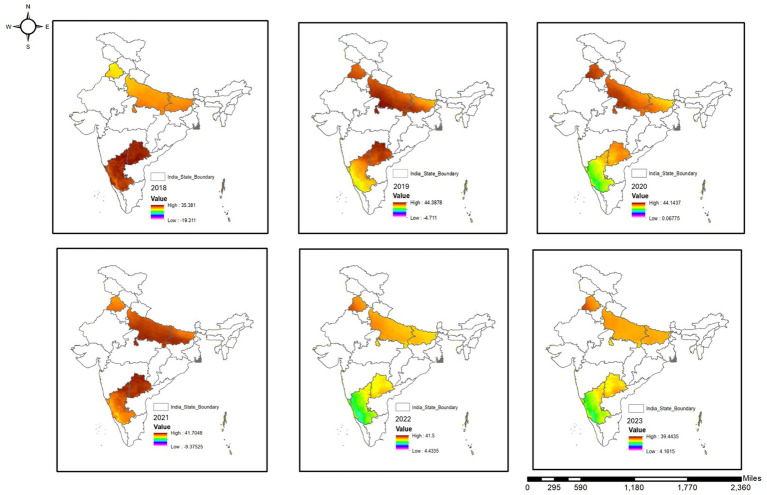
Geospatial visualization of temperature in India (2018–2023).

Figure 3 shows temperature variations across the study areas from 2018 to 2023. There are significant spatial and temporal variations. A quantitative analysis shows significant interannual fluctuations in temperature ranges: (a) 2018: 19.3 °C to 35.3 °C, (b) 2019: 4.7 °C to 44.3 °C, (c) 2020: 0.1 °C to 44.1 °C, (d) 2021: 9.3 °C to 41.7 °C, (e) 2022: 4.4 °C to 41.5 °C, and (f) 2023: 4.1 °C to 39.4 °C. The northern and central regions are highlighted in red or orange, while the southern regions are shown in green. These are short-term fluctuations, not long-term changes, indicating regional thermal instability. High temperatures in northern India and monsoon-induced cooling in the south, and consequently, human-dog interactions. This temperature variation is incorporated in the overall analysis of environmental variables and dog bite incidents. 2020 had some climatic anomalies in southern regions which has implications on ecological conditions. Meteorological fluctuations shape these trends remains to be investigated further.

### 3.2 Geospatial distribution of RH across study areas

**Figure 4:**
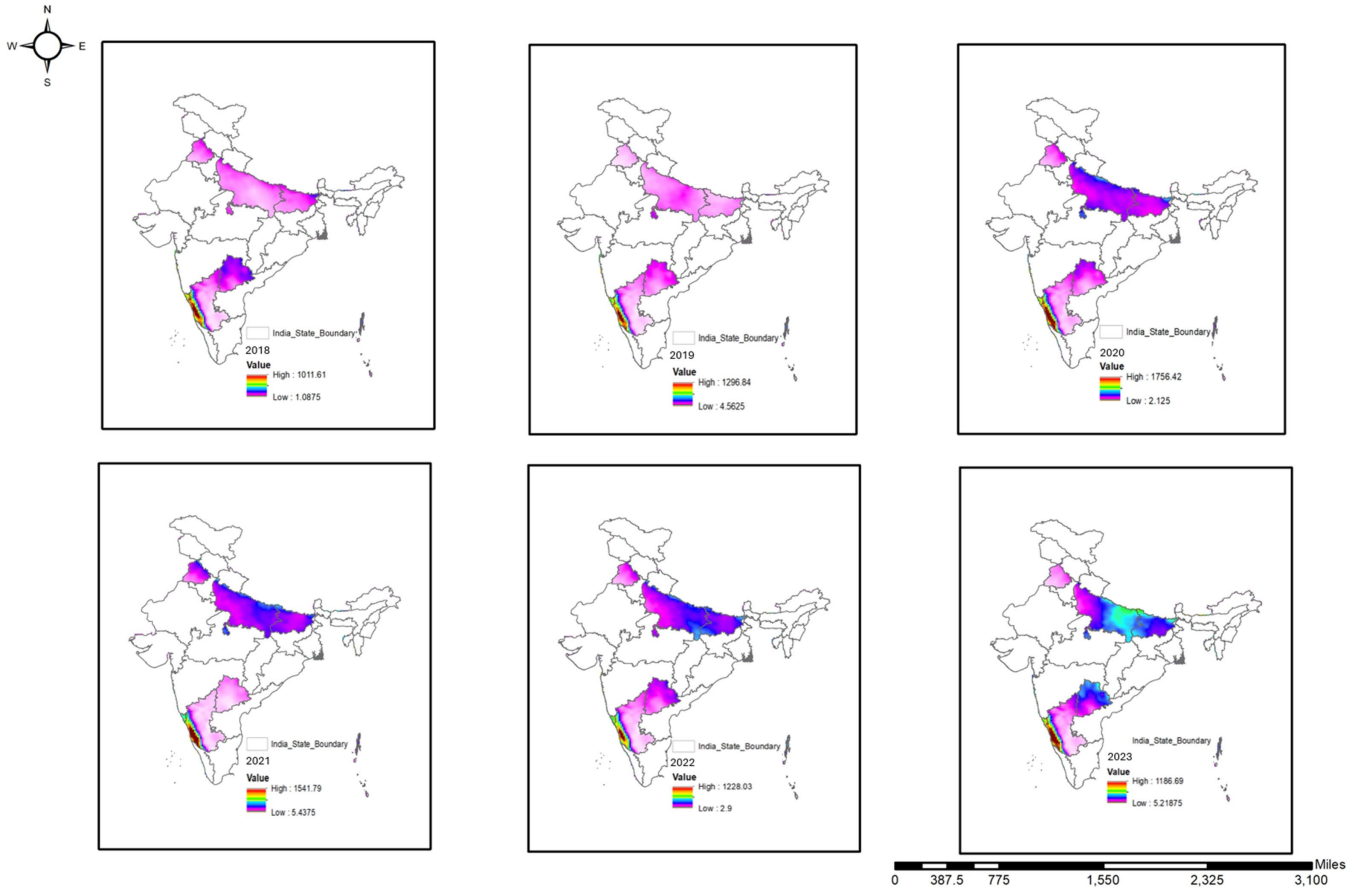
Geospatial distribution of RH across study areas (2018–2022).

The spatial analysis of RH in India from 2018 to 2022 reveals apparent spatial variability driven by climate, topography, and land use. Coastal regions, including Tamil Nadu, Kerala, and Konkan coast, which have higher RH due to proximity to water bodies, monsoon intensity, and maritime climate, as seen in Figure 4. Central and western India, namely Maharashtra, Madhya Pradesh, and Rajasthan, have lower RH due to semi-arid conditions, limited vegetation, and seasonal rainfall deficit. The Indo-Gangetic plain has intermediate RH due to irrigation, river-fed moisture, and regional weather patterns (31). Time-wise, RH trends exhibit interannual variability driven by meteorological events. In 2018, RH was climatologically normal. 2019 saw a decline across the northern and central regions due to delayed or weak monsoons. 2020 was anomalous with a sharp rise in RH over southern and eastern areas, possibly due to intensified monsoons, the Indian Ocean Dipole, and La Niña. COVID-19 lockdown-related reduction in anthropogenic emissions might have enhanced atmospheric moisture retention by reducing aerosol-induced suppression of humidity. By 2021 and 2022, RH over southern regions showed a cooling trend and was back to normal, and a localized reduction in precipitation. Central India showed high variability, possibly due to irrigation expansion and agricultural intensification, and northern regions were relatively stable. This shows that coastal proximity, vegetation cover, and monsoonal behavior are the main drivers of RH. Urban areas preeminent cities have localized humidity depressions due to urban heat island effect, reduced evapotranspiration, and land surface modification. The decline in RH in central and western India is likely due to interannual climate variability (32), but distinguishing natural from anthropogenic influences is difficult. Long-term, high-resolution studies are needed to isolate the causes and to determine how climate change and land-use dynamics affect atmospheric moisture trends (33). These spatial and temporal climate variations were mapped to establish baseline environmental conditions across states and to support subsequent statistical correlation and predictive modeling sections, where associations between dog-bite incidence and climatic factors are quantitatively assessed.

### 3.3 Analysis of dog bites in relation to climatic and pollution variables

**Figure 5:**
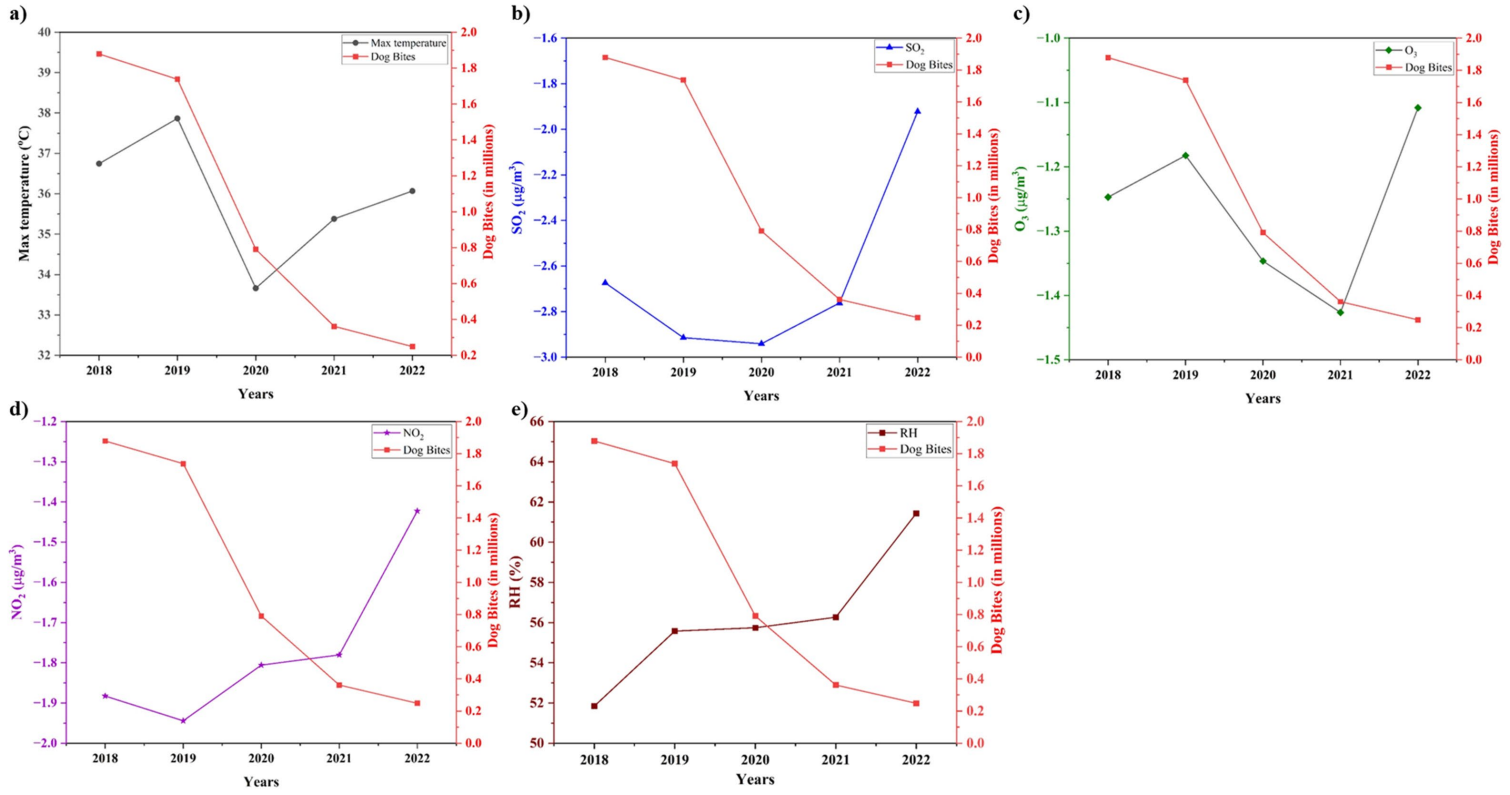
Line plot representation of the relationship between dog bites and meteorological and pollution parameters (T, RH, O₃, SO₂, NO₂) for the period 2018–2022.

Figure 5a shows non-linear relationship between maximum temperature and dog bite incidents. From 2018 to 2019, temperature increased from 36.75 °C to 37.87 °C and dog bites decreased from 1.88 to 1.74 million. Dog bites decreased significantly to 0.79 million when the temperature dropped to 33.66 °C in 2020. Despite the rise in temperatures in 2021 and 2022, dog bites continued to decrease, reaching 0.25 million in 2022. High temperatures may initially increase hostility but prolonged exposure to heat may limit dog movement or interaction, hence reducing the overall incidence. It should be noted that the visual year-to-year trend shown in Figure 5 represents aggregated annual changes, whereas the positive statistical correlation (*r* = 0.84) was derived from monthly data across all states for the complete 2018–2022 period. Therefore, the short-term nonlinear fluctuations observed in 2021–2022 do not contradict the strong, statistically significant overall positive association. Figure 5b shows that the correlation between SO₂ and dog bites is not consistent. SO₂ decreased from −2.67 μg/m^3^ in 2018 to −2.94 μg/m^3^ in 2020 when dog bites decreased from 1.88 million to 0.79 million. Despite SO₂ rising to −1.92 μg/m^3^ in 2022, dog bites continued to decline. This appears contradictory; SO₂ may not have direct or immediate effect on dog behavior but other factors such as human outdoor activity, stray dog behavior or post-lockdown conditions may be more important.

Figure 5c illustrates that O₃ levels do not correspond with the consistent reduction in dog bite incidents. O₃ decreased from −1.25 μg/m^3^ in 2018 to −1.43 μg/m^3^ in 2021, subsequently rising to −1.11 μg/m^3^ in 2022. Notwithstanding these modifications, bites continued to decline. Ozone is associated with stress reactions, but in this context it appears constrained or overshadowed by more extensive environmental or behavioral alterations. Figure 5d illustrates a comparable pattern for NO₂. Levels decreased somewhat from −1.88 μg/m^3^ in 2018 to −1.81 μg/m^3^ in 2020, and incidents of dog attacks diminished considerably. Despite the increase in NO₂ levels in 2021 and 2022, the incidence of bites continued to decline. NO₂ is not the primary driver, but it may contribute to a broader environmental background that influences behavior. Figure 5e illustrates the most distinct inverse correlation of RH. RH increased from 51.84% in 2018 to 61.43% in 2022, whereas dog bites decreased from 1.88 million to 0.25 million. Increased humidity may diminish behavior by influencing animal comfort or restricting outside interactions. Among all the variables examined, RH appears to be the most significantly correlated with a reduced incidence of dog bites. Thus, while annual trends may show non-linear behavior, the overall monthly correlation for the study period confirms an inverse association between relative humidity and dog-bite incidence.

An examination of environmental factors and dog attack statistics from 2018 to 2022 reveals some intriguing connections between dog behavior, air quality, and climate. Dog bites and RH (at 2 m) are inversely correlated till 2020 as given Supplementary Figure S1. Perhaps wetness is influencing dog behavior because lower RH is associated with more dog bites. However, this trend shifts around 2020, and we observe a rise in dog bites and RH. We must look at this more thoroughly and see how it might affect public safety regulations. NO₂ is more intricate. Reduced air pollution may be associated with decreased dog behavior because until mid-2019, a drop in NO₂ indicates consistent dog bites. Since more dog attacks occur when NO₂ levels rise after the middle of 2019, may contribute to increased dog behavior.

Pearson correlation analysis (Section 3.5) indicates statistically significant associations between dog-bite incidence and O₃ and SO₂ based on monthly paired data. However, the visual annual trend in Figure 5 does not display a uniform linear pattern. Despite fluctuations in O₃, dog bites remained consistent until 2020. Higher O₃ indicates more aggressive dogs, as both O₃ and dog bites have risen since 2020. SO₂ trends are no different. Decreased pollution is easing the problem since until mid-2019, a drop in SO₂ indicates consistent dog attacks. A rise in SO₂ after mid-2019 is associated with more dog attacks, suggesting that increased pollution may lead to more dog bite incidents. The interpretation of the SO₂ trends above refers to the visual annual patterns in Figure 5 rather than to statistical associations. Although the temporal trend does not show a yearly consistent alignment, Pearson correlation analysis in Section 3.5 identifies a statistically significant association when examined across monthly paired data.

**Figure 6:**
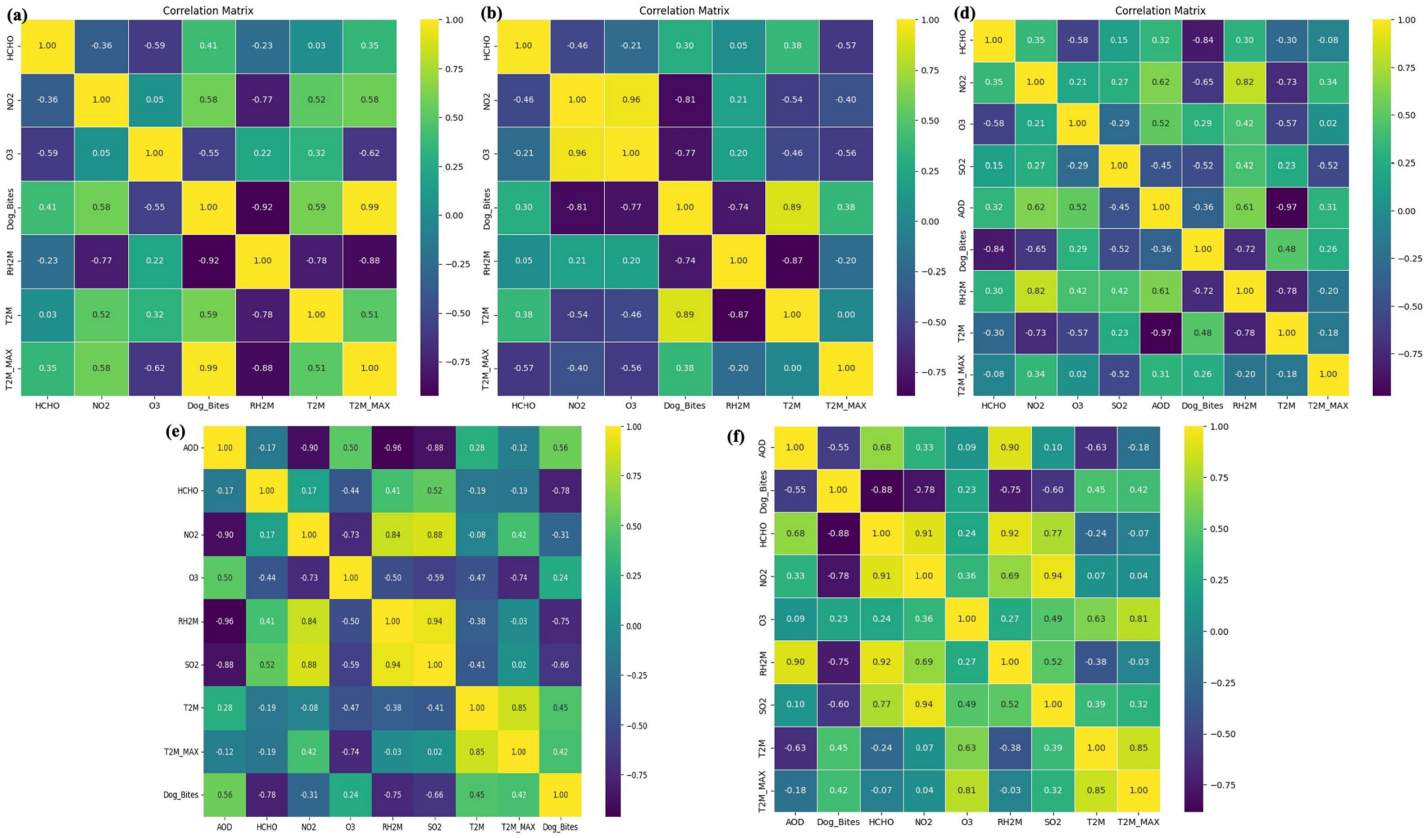
Correlation analysis of environmental factors and dog bites in the study areas (2018–2022): **(a)** Bihar; **(b)** Karnataka; **(c)** Telangana; **(d)** Uttar Pradesh.

These results demonstrate an intricate connection between dog behavior and environmental factors which comprehend the mechanisms and create focused tactics to lessen the ecological impact on dog behavior and public safety. The negative association observed visually is supported quantitatively by the Pearson correlation analysis (Section 3.5, Figure 6), which shows that RH is moderately negatively correlated with dog-bite incidence (*r* = −0.58, *p* = 0.0252). The observations in this section reflect descriptive visual trends rather than statistical conclusions. Quantitative statistical associations, including Pearson correlation coefficients and *p*-values, are presented in Section 3.5 (Correlation Matrix), which numerically evaluates the relationships between environmental variables and dog-bite incidence.

### 3.4 Environmental dynamics in Indian state: trends and implications

Between 2018 and 2022, dog bites in Bihar State showed interesting trends with respect to RH and temperature (Supplementary Figures S1a–e). The green bars are annual dog bite cases, which varied over time. Dog bite cases increased from 2018 to 2020, then decreased in 2021 and increased again in 2022, indicating that climatic, behavioral, and environmental factors are at play and warrant investigation. The blue line in Supplementary Figure S2a shows that RH was 66% in 2018 and decreased to 50% by 2020. The decrease in RH corresponds to increase in dog bites which further indicates an inverse relationship between RH and dog incidence.

Targeted measures to reduce dog bites and improve public safety requires understanding of the interplay of these variables. More research is needed to understand the impact of temperature, humidity, and other environmental factors on dog behavior in Bihar State. Between 2018 and 2022, humidity, temperature and dog bite cases in Karnataka, Punjab, Telangana, and Bihar show trends and correlations. RH in Karnataka decreased from 66% in 2018 to mid-50s in 2020 and then surpassed the initial level in 2022. RH was inversely associated with dog bites until 2021, decreased in 2020–2021, and increased in 2022 (Supplementary Figure S2). Temperature may be correlated with animal behavior as seen in the increase in dog bites in 2022. In Punjab, dog attacks peaked in 2019, decreased in 2020–2021 and increased in 2022. Like Karnataka, RH decreased till 2020 and then increased. Temperature is minimal. The inverse relationship between RH and dog bites till 2021 suggests environmental factors; but temperature seems to be of no significance. Dog bite cases in Telangana decreased from 2018 to 2022, indicating the success of interventions. RH decreased from 2018 to 2019 and then stabilized; temperature decreased till 2020 and then increased slightly. No correlation between RH or temperature and dog attacks so that other factors may be at play. Dog bite cases in Bihar decreased from 2018 to 2019 and in 2020, it was increased and stabilized. HCHO levels were increased significantly from 2019 to 2022, so suggesting that environmental or behavioral factors need to be investigated.

These studies show that the complex interplay between RH, temperature, and dog attacks. Localized studies are needed as the inverse relationship between RH and dog bites persisted in some states till 2021 despite variations. Distinct observations over redundant explanations clarify and lead to specific avoidance strategies. HCHO levels in Bihar State are increasing consistently, indicating more pollution sources. Effective pollution mitigation and public health strategies require knowledge of these sources. A gray dashed line shows NO₂ levels have a distinct pattern. After a sharp increase in 2018, NO₂ levels were decreased in 2019 and stabilized in subsequent years, indicating a need to investigate the causes of this decline. O₃ levels (orange dotted line) varied from low in 2018 to a peak in 2020/21 and then decreased slightly. Mitigation strategies are needed to control O₃ pollution in this region, as these oscillations show complex atmospheric interactions.

Dog bites and pollution in Karnataka show unusual trends. Dog bites (red line) decreased between 2019 and 2021 with pollution. Dog bites are expected to increase in 2022 despite consistent pollution. HCHO levels (blue line) increased after 2018 and inversely correlated with dog bites. NO₂ levels (green line) decreased in 2019 and then increased slightly, so dog attacks are favorable. O₃ levels peaked in 2021 and showed a moderate inverse correlation with dog bites, suggesting environmental factors may influence dog behavior. These findings demand focused public health measures. The graph of dog attacks and pollution in Punjab State from 2018 to 2022 shows complex relationships. Dog bites decreased with pollution trend in 2019 and 2021. Dog bites increased in 2022, indicating a potential public health risk. These patterns are associated with AOD which measures aerosol pollution. Telangana showed same trend. As pollution levels fluctuated from 2019 to 2021, dog bites decreased. HCHO (blue line) increased significantly after 2018 and inversely correlated with dog bites. In 2018, NO₂ (green line) peaked, significantly reduced in 2019, and then increased slightly, which may correlate with increased dog attacks. O₃ (orange line) had moderate inverse correlation with dog bites, peaked in 2021, and then decreased. Temperature (red dashed line) was 25 °C until 2021 and then an increased in 2022, coinciding with increase in dog bites. Thus, temperature may influence dog behavior. However, the environmental health implications remain complex.

### 3.5 Exploring correlations between environmental factors and dog bite incidents

Monthly averaged data on the environmental and dog bites were calculated to calculate correlation coefficients and *p*-values and identify short-term associations instead of annual summaries in the city of 20,182,022. It is necessary to highlight that these correlation coefficients are calculated from monthly data throughout the study period and should be viewed as general statistical relationships, whereas the descriptive plots in Section 3.3 show annual aggregates that may reflect local departures from the overall trend. Figures 6a–d show the correlation matrix for selected environmental variables and dog bite incidents for four Indian states. In Bihar, HCHO is negatively correlated with NO₂ (−0.36) and O₃ (−0.59) and positively correlated with dog bites (0.41). O₃ is negatively correlated with dog bites (−0.55). RH and T have the strongest associations with dog bites: Higher humidity reduces incidents, while higher temperatures increase them. In Karnataka, HCHO and NO₂ are negatively correlated (−0.46) and NO₂ and O₃ are positively correlated (0.96). O₃ is negatively correlated with dog bites (−0.77). In Karnataka, dog bites are positively correlated with RH (0.89), whereas in Bihar, RH is negatively correlated with dog bites. RH is inversely correlated with T (−0.87), whereas T and T_max_ are uncorrelated (0.00). These findings suggest that higher O₃ levels may be associated with fewer dog bite incidents, potentially due to improved air quality conditions (30).

In Telangana, AOD and NO₂ are negatively correlated (−0.90) possibly due to aerosols suppressing NO₂ concentrations. NO₂ is also negatively correlated with O₃ (−0.73) and positively correlated with SO₂ (0.88) indicating a common source of emission. RH is positively correlated with both NO₂ (0.94) and dog bites (0.94) suggesting that humidity plays a significant role in both pollutant dynamics and dog behavior. In Uttar Pradesh, HCHO is positively correlated with O₃ (0.92) and SO₂ (0.94) which indicates linked emission patterns. RH is negatively correlated (−0.75) with dog bites suggesting that increased humidity may reduce dog behavior. *p*-value analysis further highlights regional differences. In Bihar, RH (*p* = 0.0252) and T_max_ (*p* = 0.0014) are significant with dog bites while HCHO, NO₂ and O₃ are not. In Telangana, RH is significant (*p* = 0.000027), which supports the observed correlation and indicates, no significant associations were found in Karnataka or Uttar Pradesh. These findings suggest that the environmental determinants of dog bite incidents may be region-specific rather than universal. While the aggregated multi-state dataset demonstrated statistically significant correlations for certain variables, individual state-level correlation tests showed variability and were not uniformly significant across all regions, indicating regional heterogeneity.

### 3.6 PCA analysis - temperature, humidity, and dog bite incidents in Bihar

**Figure 7:**
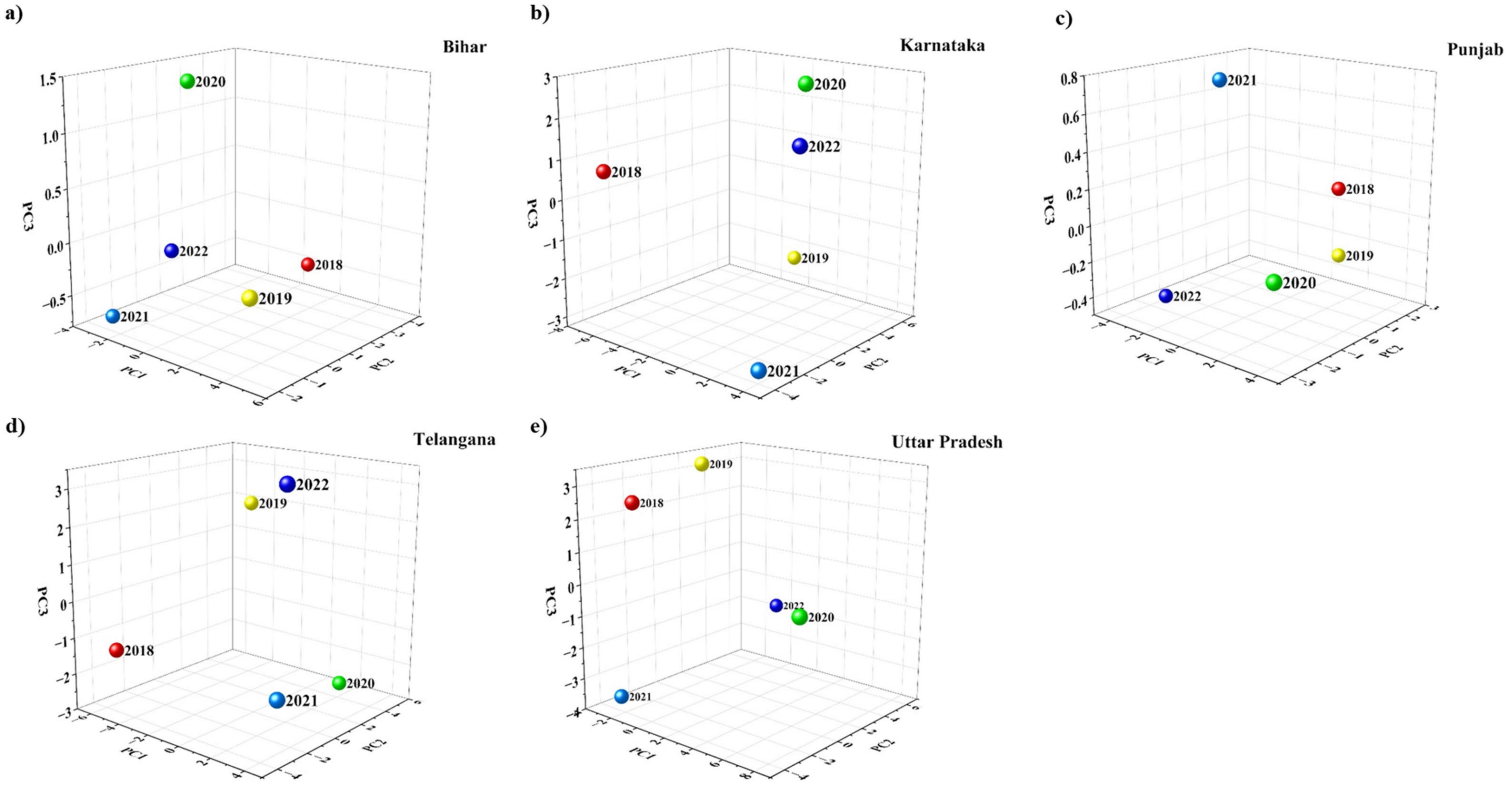
PCA analysis of dog bites, temperature, and humidity in the study areas from 2018 to 2022.

Figures 7a–e show the relationship between dog bite incidents and environmental variables (temperature and humidity) across 5 Indian states in terms of PCA. In Bihar, data points are scattered across the 3D space, with no clustering, indicating that no specific combination of temperature and humidity is consistently associated with dog bites. Some points along the PC1 axis are higher temperatures, but no trend. Similarly, points along the PC2 axis are different humidity levels but no correlation with bite frequency. Karnataka shows the same pattern. Incidents are spread all over, with no observable clusters or directional patterns. A few points along PC1 show higher temperatures during some bites, but no association. Similarly, no clear linkage is observed for humidity along PC2. In Punjab, the data points are widely dispersed on PC1, PC2 and PC3. Although temperatures are high on PC1 and humidity is fluctuating on PC2, there is no correlation with dog bite clusters. Again, randomness suggests that temperature and humidity are not the reason for bite distribution. The PCA plot for Telangana also shows scattered data with no grouping or direction. Temperatures are high on PC1 and humidity is high on PC2 but neither shows any consistent relationship with dog bite frequencies. This further supports that these two factors alone may not be enough to explain the variability. In Uttar Pradesh also the data was scattered and although some points on PC1 show high temperatures and PC2 shows humidity variation, there is no clustering or pattern. So, it seems some other variables are at play, possibly socio-economic, behavioral, or urban ecological factors. Overall, the PCA plots across all 5 states show that temperature and humidity alone do not explain dog bite patterns. Therefore, broader environmental, demographic, and behavioral factors should be considered. PCA was applied to visualize the multidimensional variance structure and the combined effects of temperature and humidity, rather than to replace correlation analysis. While only two predictors were included, PCA enabled simultaneous examination of variance loadings and dispersion patterns, supporting the conclusion that environmental variables alone do not sufficiently explain dog-bite incidence across states.

### 3.7 Prediction and modeling

Dog bite predictions were done using H2O-based Random Forest (RF) and XGBoost algorithms. Actual data showed dog bite cases peaked at 1.7–1.85 million in 2018–2019 and then declined to 0.3 million in 2022, vs. earlier estimates of 3 million. Both models captured this decline through 2020, with RMSEs below 0.2 million cases. But post 2020, the models diverged. The RF model began overestimating, predicting 0.45 million in 2021 (actual: 0.35 million) and 0.4 million in 2022 (actual: 0.3 million), with a MAPE of ~14.8%. XGBoost achieved 87% accuracy, with deviations < 0.1 million and a MAPE of 9.6%. Its RMSE of ~0.12 million was also better than RF ~ 0.18 million, indicating better generalizability beyond 2020. It was tested by 5-fold cross-validation, which produced the following mean results in RMSE 0.12 million cases (SD = 0.03) and mean MAPE 9.6% (SD = 1.8%). These values show a moderate variability but acceptable performance in considerations of the dataset size and exploratory analysis. Since few observations per state are made in a year, model predictions need to be treated with caution and as prelude to predictions and not as final ones. Incorporating behavioral and demographic factors in future datasets will improve model generalizability.

**Figure 8:**
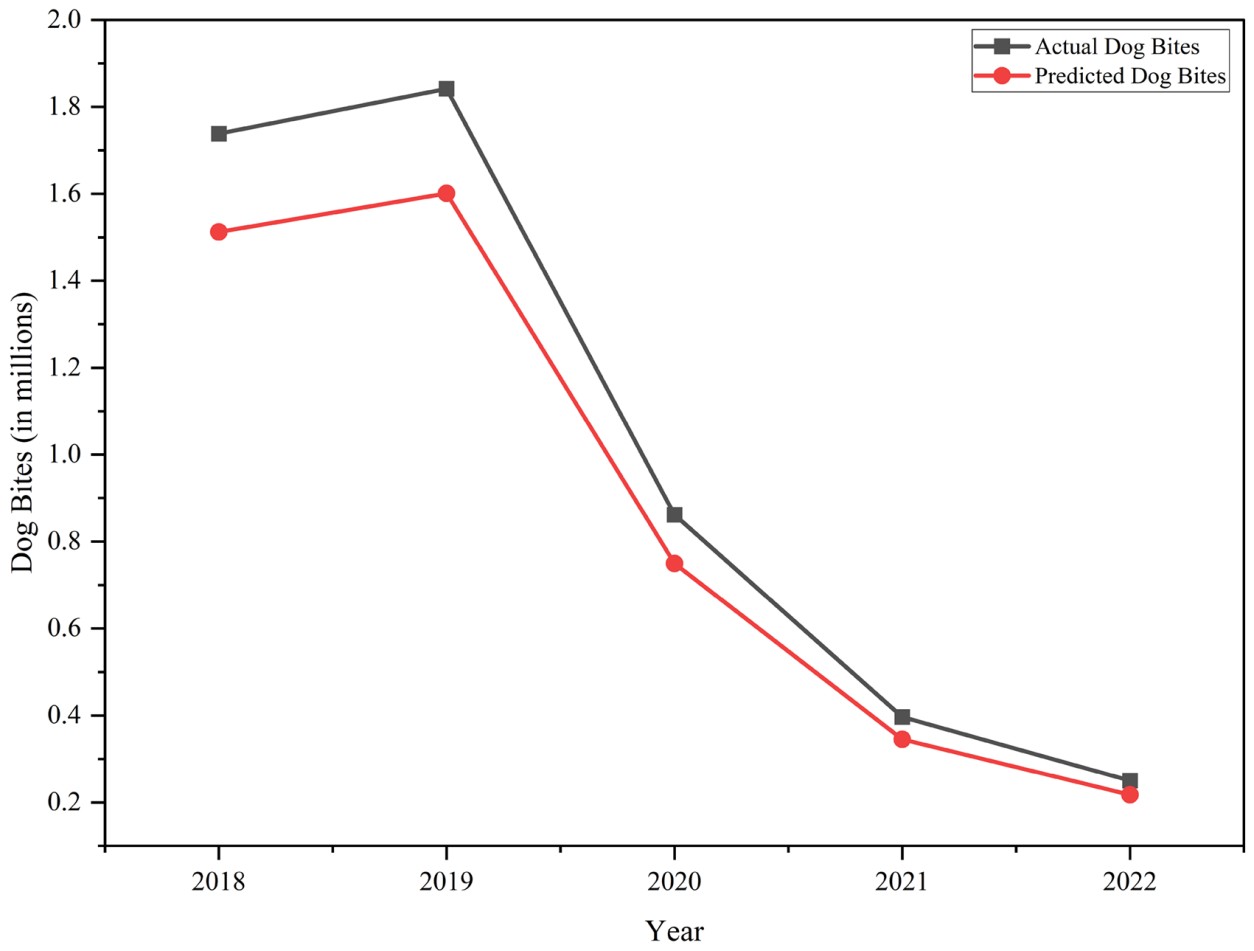
Plot depicting the comparison between actual and predicted dog bites using H2O XGBoost.

Figure 8 shows XGBoost follows the actual trend well, modeling the increase until mid-2019 and the decline after that. However, it underestimates the rate of decline post 2020 with a mean absolute error of approximately 50,000 cases in 2021 and 2022. Despite these small errors the MAPE was below 5% during this period. These errors suggest external factors like leash law enforcement, stray dog population control, and increased vaccination may have impacted real-world trends but were not captured in the models. Adding these real-world variables could improve the accuracy especially during periods of sudden change. Finally, both RF and XGBoost performed well in tracking dog bite trends but XGBoost performed better in stability, precision, and adaptability. With 87% overall accuracy XGBoost is a good tool for forecasting dog bite cases and guiding targeted public health interventions. It should be noted that the temporal decline in dog bites from 2019 to 2022 was significant, which could be a major contributor to the model’s performance, and the model may have been partially learned from the structural trend rather than solely influenced by climatic and pollutant data. Several major confounding variables like dog population control activities, vaccination campaigns, and alterations in the outdoor mobility of the people were not available in this data and therefore not factored. Due to this, predictive accuracy must not be understood as a causal explanation of environmental drivers, instead it is a forecasting ability.

## 4 Discussion

The Discussion presents exploratory interpretation of patterns observed, intended to generate hypotheses rather than assert causal relationships, given the ecological and preliminary nature of the dataset. This study investigated the relationship between dog bite incidents and environmental factors such as air pollutants and climate across four Indian states (Bihar, Punjab, Telangana, and Uttar Pradesh) from 2018 to 2022. The correlation matrices reveal substantial spatial variability, demonstrating that temperature, humidity, and pollutant effects operate differently across states. Therefore, the study does not claim a single universal directional relationship but rather highlights the need for region-specific strategies. The results show regional variation, due to a mix of ecological, environmental, and socio-behavioral factors. In Bihar dog bite incidents fluctuated over time, HCHO levels went up steadily, indicating a persistent pollution problem. NO₂ levels decreased, possibly due to improved air quality regulations. O₃ levels varied, showing complexity of atmospheric chemistry. These opposite trends highlight the need to identify pollutant-specific impact on public health and develop strategies that account for both direct and indirect environmental factors.

Based on exploratory observations, pollutants such as NO₂, O₃, HCHO, and SO₂ may show variable associations with dog-bite incidence in Punjab. However, these trends were not statistically validated in this study and should be interpreted cautiously. Presence of strong pollutant-dog bite associations in Punjab means regional interventions that combine pollution reduction with public safety measures are needed. In Telangana, the relationship between dog bites and environmental parameters was more complex. NO₂, O₃ and HCHO had significant correlations, temperature and humidity did not. Exploratory trend comparison suggested potential inverse behavior between O₃ levels and dog-bite trends; however, this was not supported by a consistent pattern in the Results and should be interpreted as a hypothesis for further investigation rather than a confirmed finding. Accordingly, the pollutant–dog bite patterns described for Punjab and Telangana should be regarded as exploratory and non-causal, and no strong associations are inferred beyond the statistically supported correlations reported in Section 3.5. The state’s climate and urban landscape may be influencing these patterns, so localized, multi-factorial analysis is needed. The contrasting results across states indicate that environmental determinants of dog bite incidence operate differently across local climatic, ecological, and administrative contexts, rather than reflecting a single national trend.

In Uttar Pradesh, dog bite trends were influenced by a combination of pollutants (NO₂, O₃, SO₂, HCHO) and RH at 2 meters (RH2M). PCA showed scattered data with no clustering, so environmental variables do not explain the bite patterns. Broader factors like population density, human-dog interaction frequency and regional habits must be playing a big role. *p*-value analysis further showed regional variations. None of the 5-temperature related *p*-values were below 0.05, but one (0.0852) was close. This shows the complexity of environmental factors on dog bites and we need more granular state level studies with additional behavioral and socio-economic variables. These regional variations are likely due to differences in urbanization, stray dog population, pollution, public health infrastructure and dog culture. The lack of statistically significant associations in Uttar Pradesh reinforces the idea that environmental factors alone may not consistently explain dog-bite trends across states and supports the need for more granular analyses incorporating behavioral and socio-economic determinants. Data collection standards and reporting accuracy may also vary. So future studies should have stratified regional analysis and add more layers like behavioral data, urban design metrics and socio-economic indicators to explain local patterns. H2O XGBoost predictive model achieved 87% accuracy in predicting dog bite incidents in Telangana for 2023 with RMSE and MAE of 3.45. Though the performance was good, we missed some critical predictors like stray dog population data and human behavioral variables. The model did not capture sudden behavioral or environmental changes well, so we need to add more input features for future iterations.

The study evaluated simpler models such as Linear Regression, Lasso Regression, and Decision Trees; nonetheless, it exhibited significantly greater errors. Compared to Linear Regression, H2O XGBoost reduced error by more than 80%, making it suitable for high-dimensional, nonlinear problems. Random Forest effectively identified early trends, but was surpassed by XGBoost in subsequent years due to its superior generalization and regularization abilities. XGBoost effectively models intricate relationships among contaminants, climate variables, and behaviorally influenced outcomes, rendering it appropriate for this investigation. Its utilization in further intricate environmental prediction tasks, such as wind energy forecasting, serving as a proof of concept (31). The lack of external validation due to the dataset’s uniqueness is a drawback. Subsequent research should investigate multi-source validation and further model refinement using behavioral and demographic data to enhance predictions. The observed high predictive accuracy of XGBoost reflects the dominant temporal pattern in the data, suggesting that incorporating additional confounders and policy-based variables is essential for improving causal interpretation in future studies.

## 5 Conclusion

This study investigated the dog bite incidents in 5 Indian states namely, Bihar, Karnataka, Punjab, Telangana, and Uttar Pradesh between 2018 and 2022. Complex associations between dog bites, air pollutants, and environmental conditions were also analyzed. The study identified state-wise differences in the associations between meteorological and pollution factors and dog bite incidence, with temperature and relative humidity showing significant relationships in specific regions, while pollutant effects did not follow a consistent national trend. In particular, the statistical analysis showed that RH (*p* = 0.0252) and maximum temperature (*p* = 0.0014) were statistically significant in Bihar and Telangana.

To explore the predictive potential, H2O XGBoost algorithm was used and it achieved 87% accuracy. It performs well in modeling, non-linear, multi-dimensional environmental data. The model captured the historical trends and provided reasonably accurate forecasts. Making it a useful tool for proactive public health planning. But some deviations in recent years suggest that we need to include additional variables like population density, stray dog count, human outdoor activity, and dog behavior pattern to improve the prediction accuracy.

Notably, the results align with and support various United Nations Sustainable Development Goals. Preventing rabies and injuries, the article also directly impacts SDG 3 (Good Health and Well-being). SDG 11 (Sustainable Cities and Communities) is supported by its focus on addressing risks in densely populated urban and peri-urban areas. In addition, the research also offers practical measures with an eye to SDG 13 (Climate Action) given that the study exposes the effect of climatic and pollution-related stressors on the human-animal conflict.

The findings highlight the need for region-specific approaches to dog bite prevention as environmental factors vary across states. A uniform policy framework may not work for local dynamics. Instead, tailored interventions based on ecological, social, and behavioral data can be implemented to reduce risk and increase public safety. These findings will help future studies develop adaptive, data-driven strategies that incorporate real-time behavioral and ecological indicators for dog bite management across varying climatic and urban settings. The study did not implement multivariate regression or adjust for confounding variables due to a lack of consistent datasets across states; these will be incorporated in future research to strengthen causal inference.

## Data Availability

The original contributions presented in the study are included in the article/supplementary material, further inquiries can be directed to the corresponding author/s.
